# Editorial: Decision-making and planning for multi-agent systems

**DOI:** 10.3389/frobt.2024.1422344

**Published:** 2024-05-24

**Authors:** Panagiotis Tsiotras, Matthew Gombolay, Jakob Foerster

**Affiliations:** ^1^ School of Aerospace Engineering, Georgia Institute of Technology, Atlanta, GA, United States; ^2^ School of Interactive Computing, Georgia Institute of Technology, Atlanta, GA, United States; ^3^ Department of Engineering Science, Oxford University, Oxford, United Kingdom

**Keywords:** multi-agent systems, robotics, reinforcement learning, decision-making, planning

Multi-agent systems are widely applicable to real-world applications ranging from warehouse automation to environmental monitoring, autonomous driving, and even computer game simulations. Compared to single agents, coordinated multi-agent systems have greater potential to tackle time-sensitive, complex, and large-scale problems. However, orchestrating the behaviors of multi-agent systems for cooperative or non-cooperative tasks is a difficult computational optimization problem. Even though decision-making and reinforcement learning (RL) techniques for single-agent scenarios have seen tremendous achievements in recent years, we have not yet seen a widespread translation of these single-agent techniques to the multi-agent domain. Unfortunately, translating single-agent techniques to the multi-agent setting is not straightforward, and many challenges exist, stemming primarily from the intrinsic nature of the multi-agent systems, including complex interaction dynamics, constrained inter-agent communication, various notions of optimality, heterogeneity, as well as the potential presence of adversaries.

The objective of this Research Topic is to report on the recent advances in multi-agent planning and decision-making. While decision-making and planning for a single agent has been extensively studied, the multi-agent version of the problem has not received the same attention in the literature and is much less understood. In addition to sensing and planning challenges common to the single and multi-agent settings, multi-agent systems must also deal with the additional requirements of communication and coordination among the agents. Further, several multi-agent architectures assume very large numbers of agents, each having limited computational resources. Decentralized architectures that consider these limitations are essential for most practical applications, such as in low-size, -weight, and -power (low-SWaP) settings. Moreover, coordination and cooperation among the agents can be either altruistic or individualistic, and deciding between these two options (and when) is not straightforward.

By reporting on the latest advances in the field, this Research Topic aims to make the community aware of the existing challenges of the multi-agent decision-making problem, and disseminate recent and novel research trends in this area.

This Research Topic includes five papers spanning a variety of themes related to multi-agent decision-making. We overview these papers below.

First, one of the main challenges in multi-modal learning for one or more agents is finding a way to create a shared representation of different data types (i.e., modes) without explicit feedback between the agents. In the paper *“Emergent communication of multimodal deep generative models based on Metropolis-Hastings naming game,”* Hoang et al. address the problem of two agents jointly observing a shared subject with the objective of developing a common vocabulary. This is an instance of the so-called “emergent communication” (EmCom) where the agent incorporates multimodal information to enrich learning by providing multiple viewpoints on a dataset for a more accurate and robust communication strategy. By leveraging information from multiple sources, such as visual, auditory, and textual data, it is expected that a deep generative model can capture and exploit the complementary nature of the different data types. The authors propose a model for emergent communication of multimodal deep generative models of two agents that combines a Gaussian mixture model (GMM), a multimodal variational autoencoder (MVAE) and a Metropolis-Hastings (MH) naming game to form perceptual categories and exchange signs between the two agents within multimodal contexts. The MH naming game is a language game played by two agents, where one agent (the speaker) observes an object and names it based on its perception and communicates a word (i.e., a sign) by choosing from a posterior word distribution related to the object. The second agent (the listener), decides whether to accept the sign based on its own understanding, and the process is repeated by switching the roles between the two agents. The experimental results on the MNIST + SVHN and Multimodal165 datasets demonstrate that combining the Gaussian mixture model (GMM), PoE multimodal VAE, and MH naming game substantially improved information sharing, knowledge formation, and data reconstruction.

Second, *“Reactive optimal motion planning for a class of holonomic planar agents using reinforcement learning with provable guarantees”* by Rousseas et al. addresses the classical problem of planning for holonomic planar robotic agents. Most existing methods have been based on trajectory optimization techniques that provide optimality guarantees but are computationally expensive. On the other hand, reactive methods provide robust, provably convergent solutions but often lack optimality guarantees. These authors utilize ideas from RL to address the limitations of reactive methods. A policy iteration RL scheme is employed to construct the optimal input without necessitating the solution of an intractable nonlinear partial differential equation. In addition, safety, convergence, and policy improvement are guaranteed using rigorous control-theoretic arguments. Using numerical examples, it is shown that the proposed method outperforms, or closely matches, state-of-the-art planning methods such as PRM or RRT*, while readily providing a solution for all initial conditions in the workspace.

Third, *“Decentralized multi-agent reinforcement learning based on best-response policies”* by Gabler and Wollherr also employ RL concepts to find optimal policies for multi-agent systems. The authors propose an actor-critic (AC) approach for cooperative multi-agent RL (MARL) problems in sparsely rewarded domains. The proposed approach decouples the MARL problem into a set of distributed agents that model the other agents as responsive entities. Two separate critics per agent are used so as to distinguish between the joint task reward and the agent-based costs. For the joint team reward, and since the critic depends on the joint action of all agents, two models are proposed based on the theory of Stackelberg games: a game against nature, and a dyadic game against each agent. As a result, the proposed algorithm leads to fully decentralized execution and training, outperforming other competing MARL methods.

The last two papers both deal with the ubiquitous problem of cooperative robot navigation. In the paper ‘*‘Terrain-aware semantic mapping for cooperative subterranean exploration,”* Miles et al. address the problem of mapping in challenging subterranean environments. The authors of that paper propose a modular framework for semantic mapping of such subterranean environments. The approach uses occupancy and traversability information encoded in a grid map, which is then distributed amongst the team robots, while also respecting the limited bandwidth constraints. The approach is validated experimentally using both high-fidelity simulations as well as physical experiments. As a matter of fact, the proposed multi-agent mapping algorithm was implemented on Team MARBLE’s entry in the DARPA Subterranean Challenge, where it received third place.

Finally, the paper *“Cooperative planning for physically interacting heterogeneous robots”* co-authored by Sebok and Tanner proposes an approach to solve the problem of cooperative behavior planning for small heterogeneous teams of robots, where the members of the team can physically interact with each other, for instance, one robot can push, the other can pull or lift, etc. In many applications, such robot heterogeneity is essential to accomplishing a task that is not possible using a single robot (or even a team of robots) with a single modality. To solve this challenging problem the authors introduce a hybrid automaton to model modality transitions and then use hybrid dynamical systems theory to capture the full closed-loop dynamic of the robot team. The combined heterogeneous multi-robot system planning and control architecture is capable of expressing cooperative group behaviors that are quite distinct from those of its group members. The approach is tested on two case studies, one tethered UAV–UGV system, and the other a dual UGV system in which the two UGVs differ owing to their different locomotion modalities and motion degrees of freedom.

The five contributions to this Research Topic offer an informative and broad perspective on the complexity of multi-agent systems, highlighting some of the inherent challenges and how to address them. Specifically the five papers address important issues related to communication (Hoang et al.), cooperative navigation (Sebok and Tanner and Miles et al.), and RL approaches in multi-agent settings (Rousseas et al. and Gabler and Wollherr). Of course, this is an active area of research. The findings in these papers offer advances in the current state of knowledge, and, at the same time, invite further research in the area of multi-agent decision-making, suggesting several potential extensions.

